# Supplementary material for: Endosomal PI(3)P regulation by the COMMD/CCDC22/CCDC93 (CCC) complex controls membrane protein recycling
Source: Nat Commun. 2019 Sep 19;10:4271. doi: 10.1038/s41467-019-12221-6 (PMC6753146; doi:10.1038/s41467-019-12221-6)
Supplement: Supplementary file 1 — Supplementary Information [file 41467_2019_12221_MOESM1_ESM.pdf]

Supplementary information

# Endosomal PI(3)P regulation by the COMMD/CCDC22/CCDC93 (CCC) complex controls membrane protein recycling

Singla, Fedoseienko et *al., 2019*

# Supplementary figure 1

a

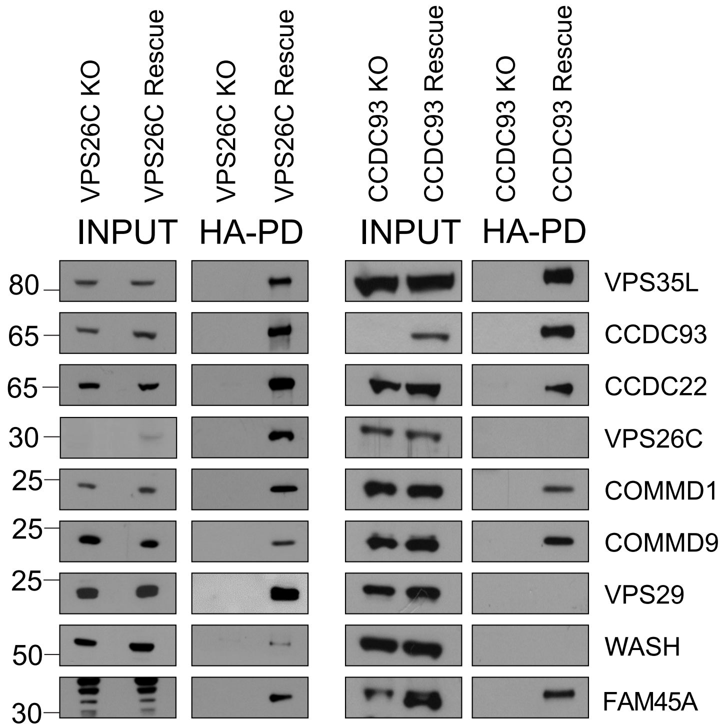


b

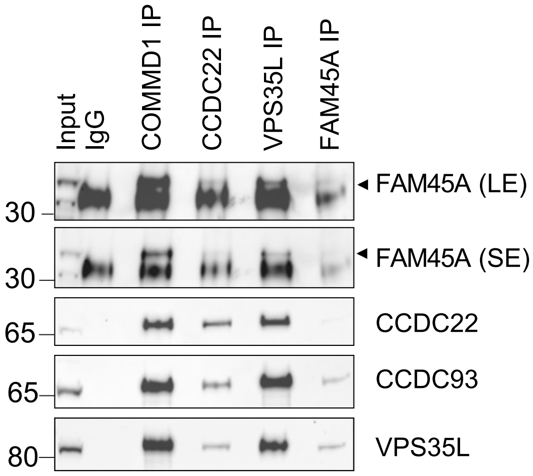


c

| CCC component | Relative representation |
|---|---|
| COMMD10 | 100% |
| COMMD6 | 100% |
| COMMD8 | 100% |
| FAM45A | 100% |
| COMMD1 | 87% |
| CCDC22 | 70% |
| COMMD2 | 70% |
| COMMD3 | 70% |
| COMMD7 | 67% |
| COMMD9 | 67% |
| COMMD4 | 65% |
| VPS29 | 65% |
| CCDC93 | 52% |
| KIF2B | 44% |
| VPS35L | 43% |
| COMMD5 | 43% |
| VPS26C | 35% |
| FAM167A | 31% |
| E2F6 | 25% |
| TRIM16L | 25% |
| CTAGE5 | 19% |
| CCDC151 | 16% |
| TFDP1 | 16% |
| VPS33B | 12% |

d

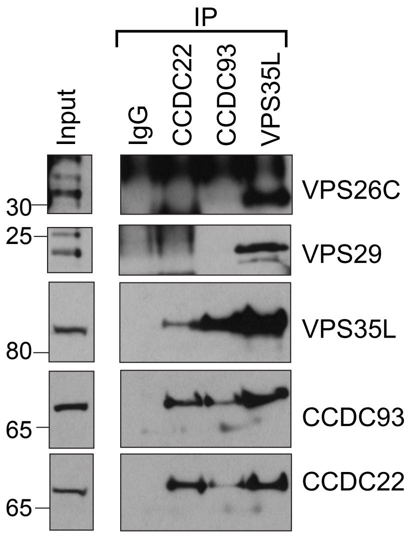

**Supplementary figure 1.** CCC and Retriever interactome analysis. **(a)** Immunoprecipitation and immunoblotting in VPS26C and CCDC93 CRIPSR/Cas9 KO lines, which were then stably transduced with an empty vector (EV) or HA-tagged versions of VPS26C or CCDC93. Immunoprecipitation of VPS26C or CCDC93 from cell lines was followed by immunoblotting for the indicated proteins. Representative of 2 independent iterations. **(b)** Immunoprecipitation of the indicated proteins (COMMD1, CCDC22, VPS35L, FAM45A) from HEK293T cell lysates, followed by immunoblotting. LE, long exposure; SE, short exposure. Arrows point to the band that corresponds to FAM45A, seen above the IgG light chain region. **(c)** Interactome results for components of CCC or retriever in previously reported protein-protein interaction studies in human cells and *Drosophila*. The number of times that any indicated protein was identified, was tabulated and normalized by the maximum theoretical occurrences in the examined gene matrix, which is presented in the form of percentage. **(d)** Immunoprecipitation of endogenous CCDC22, CCDC93 and VPS35L was followed by immunoblotting for the indicated proteins (representative of at least 3 iterations).

# Supplementary figure 2

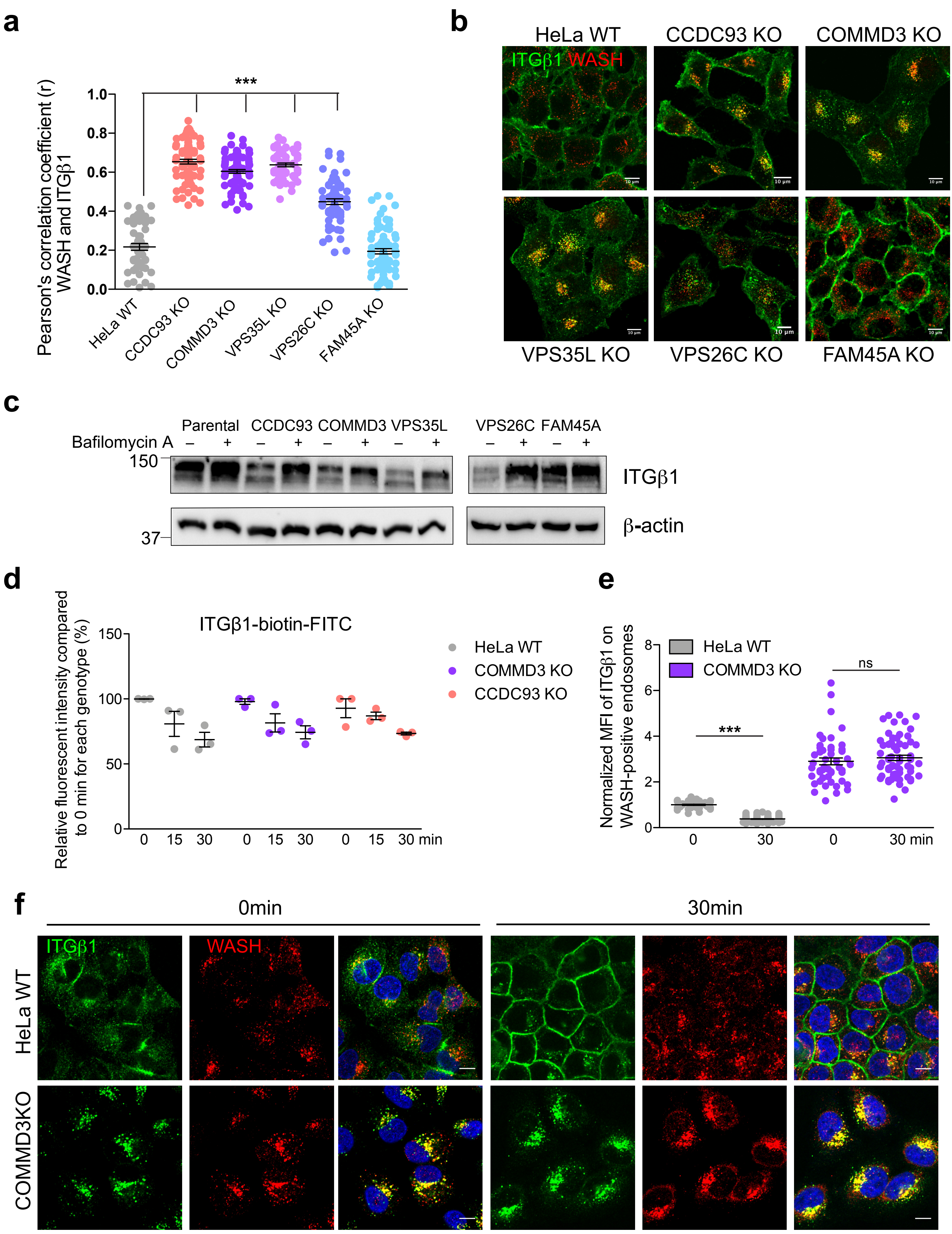

**Supplementary figure 2.** CCC deficiency leads to defective ITGβ1 recycling. **(a)** Pearson correlation coefficients for WASH and internalized integrin β1 (ITGβ1) following a recycling assay. Results for individual cells are plotted, along with the mean and s.e.m for each group (n=50 cells for WT, 75 for CCDC93 KO, 79 for COMMD3 KO, 57 for VPS35L KO, 61 for VPS26C KO, 77 for FAM45A KO); ****P*<0.0001 (one-way ANOVA and Dunnett's test to control). **(b)** Confocal microscopy imaging of ITGβ1 and WASH immunofluorescence staining quantified in (a). Representative images of 2 independent experiments are shown. Scale bars, 10 µm. **(c)** Knockout and parental HeLa cells were incubated with vehicle or lysosomal inhibitor Bafilomycin A prior to lysis. ITGβ1 protein levels analysed by western blotting. Representative blots from 3 independent experiments. **(d)** ITGβ1 endocytosis dynamics were determined by staining surface ITGβ1 in the indicated cell lines, using ITGβ1-biotin antibody. Internalization rate of ITGβ1-biotin-FITC was measured by FACS analysis (as described in the methods section) and normalized to 0 minutes time point for each genotype. The results are plotted as the mean and s.e.m for each group. Representative of 3 independent experiments. **(e)** Recycling of ITGβ1 was determined in parental and COMMD3 KO cells following ITGβ1 surface pulse labeling and subsequent stripping of remaining surface antibody (as described in the methods section). Normalized mean fluorescent intensity for ITGβ1 on WASH-positive areas was quantified. The results are plotted as the mean and s.e.m for each group (n=36 cells for WT 0 minutes, 107 for WT 30 minutes, 48 for COMMD3 KO 0 minutes, 60 for COMMD3 KO 30 minutes); ***P<0.0001; not significant, ns (unpaired two-tailed t-test to 0 minutes). **(f)** Confocal images depicting the subcellular localization of ITGβ1 and WASH in the recycling experiment quantified in (e) are shown. Representative images of 2 independent experiments. Scale bars, 10 µm.

# Supplementary figure 3

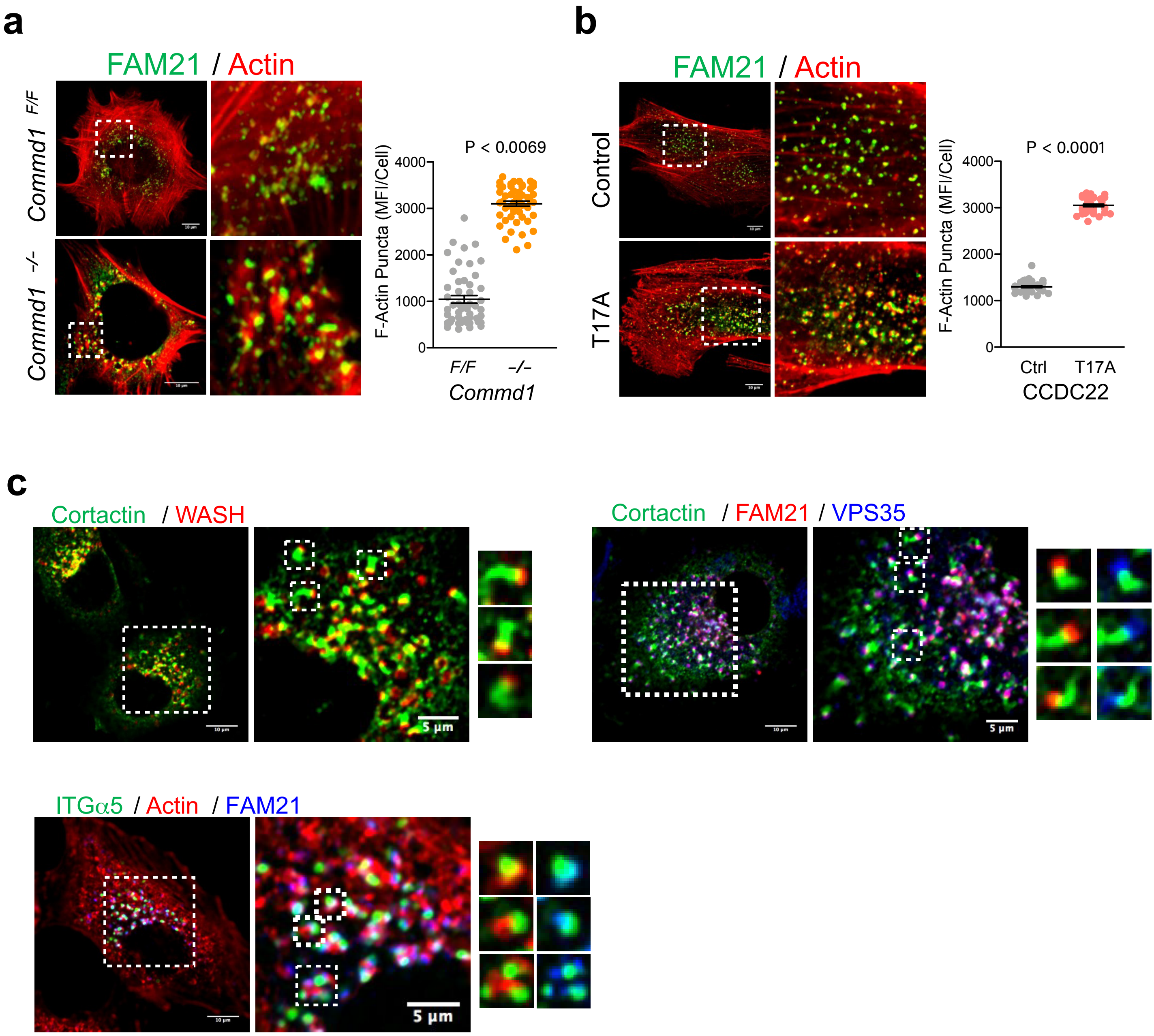


**Supplementary figure 3. Cortactin and actin form comets in COMMD3 KO cells. (a)** Representative fields of view from F-actin and WASH immunofluorescence staining in COMMD1 knockout (*Commd1*$^{-/-}$) mouse embryonic fibroblasts compared to control cells (*Commd1*$^{F/F}$). Scale bars, 10 μm**.** Mean fluorescence intensity of F-Actin on FAM21-positive vesicles was quantified in the indicated cell lines (n=50 cells). The results are plotted as the mean and s.e.m for each group (unpaired two-tailed t-test to control). **(b)** Representative fields of view from F-actin and WASH immunofluorescence staining in CCDC22 p.T17A fibroblasts compared to control fibroblasts. Scale bars, 10 μm**.** Mean fluorescent intensity of F-Actin on FAM21-positive vesicles was quantified in the indicated cell lines (n=34 cells). The results are plotted as the mean and s.e.m for each group (unpaired two-tailed t-test to control). **(c)** Confocal images of COMMD3 KO cells showing presence of WASH, FAM21 and VPS35 at the tips of cortactin tails, and ITGα5 with FAM21 at the tips of actin tails. Scale bars, 10 μm; 5 μm on zoomed images.

# Supplementary figure 4

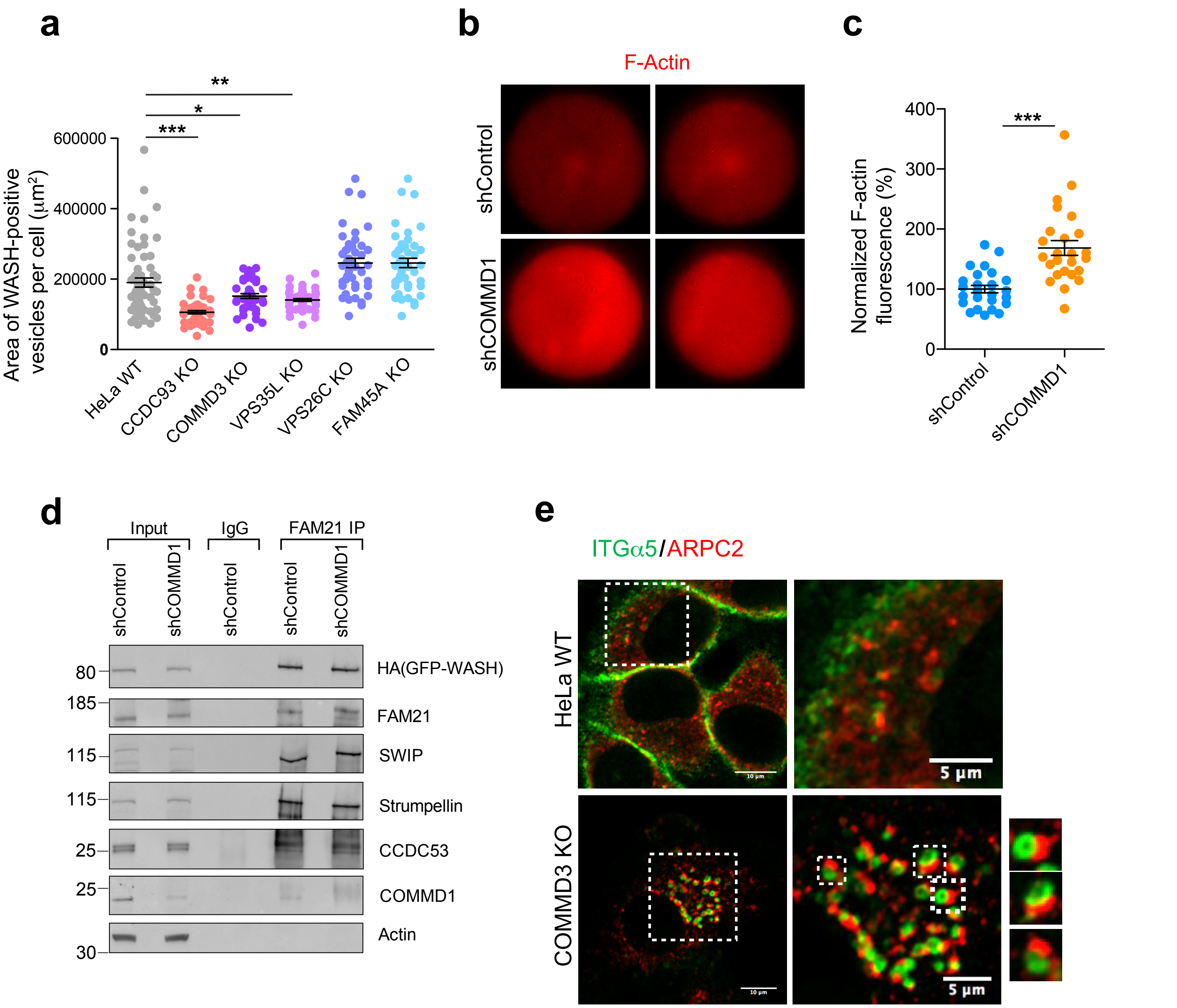


**Supplementary figure 4. WASH activity and distribution is altered in CCC-deficient cells. (a)** WASH-positive area was quantified (n=62 cells for WT, 45 for CCDC93 KO, 48 for COMMD3 KO, 50 for VPS35L KO, 43 for VPS26C KO, 48 for FAM45A KO) and plotted as the mean and s.e.m; ****P*<0.0001; **P<0.005; *P<0.05 (one-way ANOVA and Dunnett's test to control). Representative images are shown in Figure 4b. **(b)** Representative images of agarose beads following in vitro F-actin polymerization in the indicated cell lines. The WASH complex was immunoprecipitated using a FAM21 antibody and incubated with G-actin and Arp2/3 complex. **(c)** Bar graph representation of the mean and s.e.m for F-actin fluorescence in (b); n=25 beads were quantified per group; ***P<0.0001 (unpaired two-tailed t test to control). **(d)** FAM21 was immunoprecipitated from HA-GFP-WASH shControl and shCOMMD1 cell lysates followed by immunoblotting for the indicated proteins. Actin serves as a loading control. **(e)** Representative confocal images of HeLa WT and COMMD3 KO cells costained for internalized ITGα5 and ARPC2. Scale bars, 10 μm; 5 μm on zoomed image.

# Supplementary figure 5

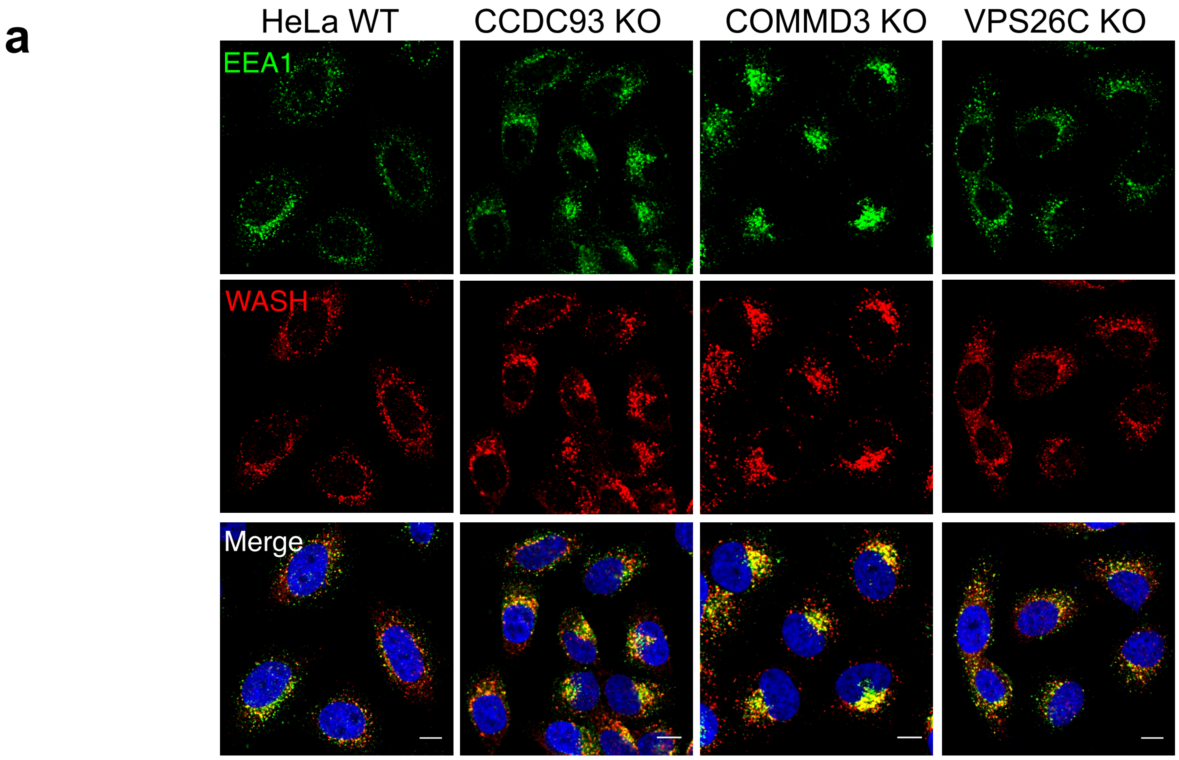


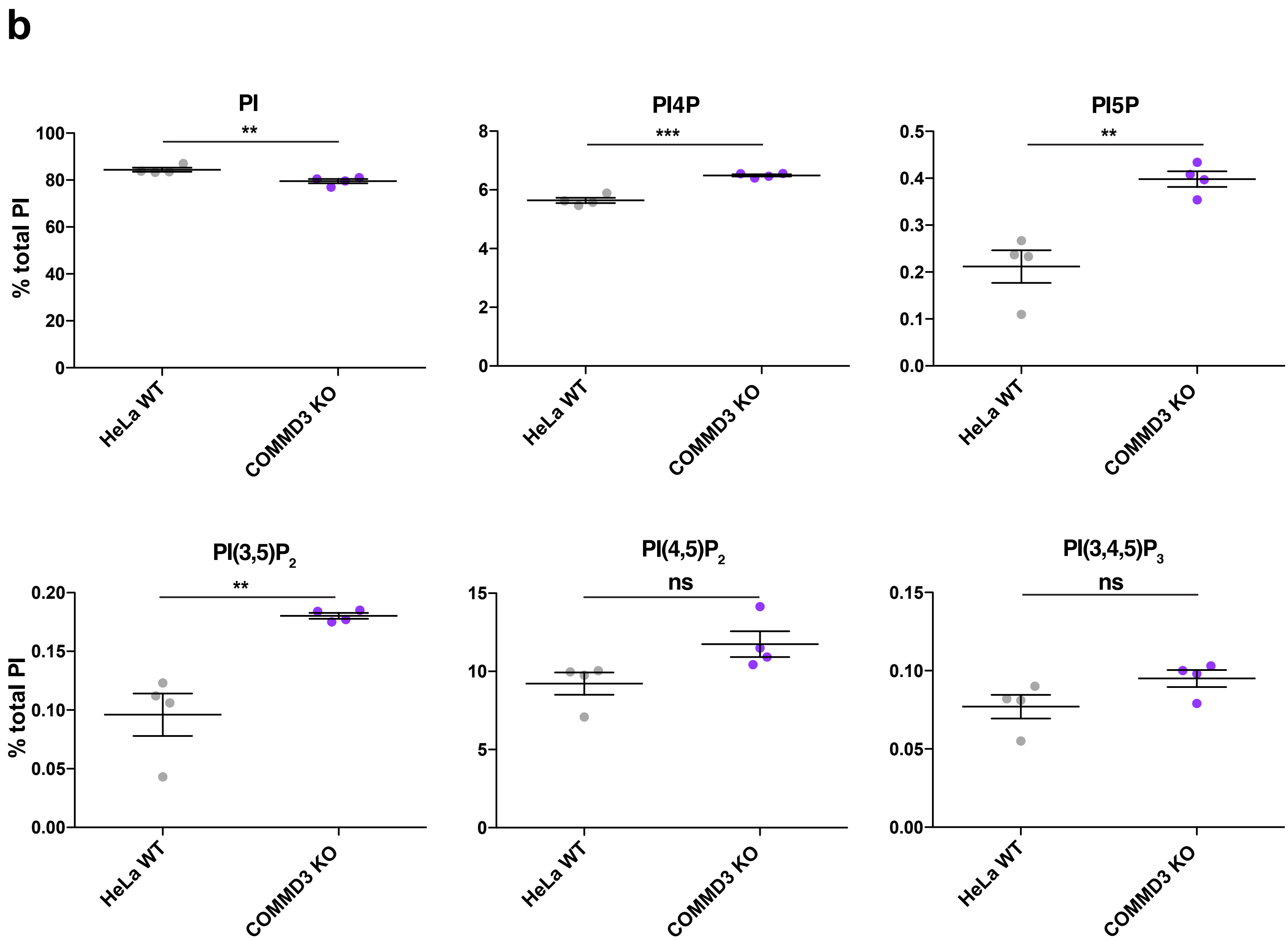

**Supplementary figure 5. The levels of PI(3)P, PI(4)P and PI(3,5)P2 are increased in CCC-deficient cells. (a)** Representative fields of view from EEA1 and WASH immunofluorescence staining experiment partially shown in Figure 5a. Scale bars, 10 µm. **(b)** Phosphorylated phosphoinositide levels were quantitatively measured in HeLa WT and COMMD3 KO cells by HPLC as described in the methods section. PI levels were significantly decreased (**p=0.0083), while PI(4)P (***p=0.0001), PI(3,5)$P_2$ (**p=0.0036) and PI(5)P (**p=0.0029) levels were significantly elevated in COMMD3 deficient cells as compared to HeLa WT. PI(4,5)$P_2$ and PI(3,4,5)$P_3$ levels were not significantly changed (p=0.0593 and p=0.1024, respectively). The results are plotted as the mean and s.e.m for each group. Statistical significance was analyzed using unpaired two tailed student t-test (n = 4 biological replicates).

# Supplementary figure 6

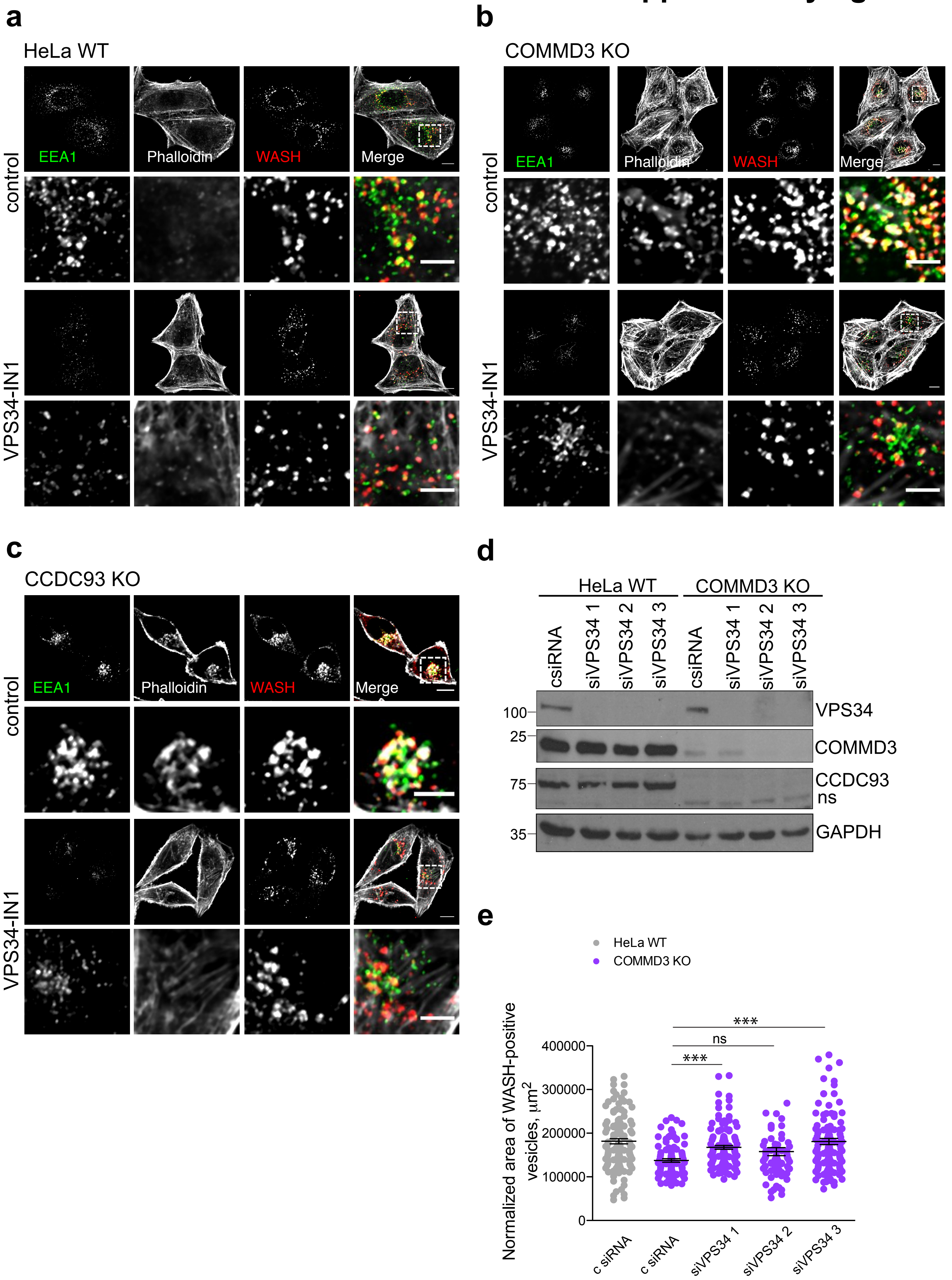

**Supplementary figure 6. Accumulation of actin in CCC KO cells is PI(3)P-dependent. (a), (b), (c).** Representative confocal images for EEA1, phalloidin (F-Actin), and WASH in WT (a), COMMD3 KO (b) and CCDC93 KO (c) HeLa cells treated with vehicle control or VPS34 inhibitor VPS34-IN1 (1 μM) for 1 h. Scale bars, 10 μm, zoom 5 μm. Normalized phalloidin and WASH fluorescence on EEA1-positive areas was quantified in Figure 6b. **(d)** HeLa WT and COMMD3 KO cells were treated with control siRNA or 3 independent siRNAs against VPS34 for 72 hours, cells were lysed, and levels of the indicated proteins were analyzed by immunoblot. **(e)** WASH-positive area was quantified in parental and COMMD3 KO cells, transfected with either control or 3 independent siRNAs targeting VPS34. Results for individual cells are plotted, along with the mean and s.e.m for each group (n=130 cells for HeLa WT control siRNA (csiRNA), 90 for COMMD3 KO csiRNA, 120 for COMMD3 KO siVPS34 1, 85 for COMMD3 KO siVPS34 2, 130 for COMMD3 KO siVPS34 3); ***$P<0.0001$, ns=not significant (one-way ANOVA and Dunnett's test to COMMD3 KO control siRNA). For representative images of WASH staining please see Figure 6c.

# Supplementary figure 7

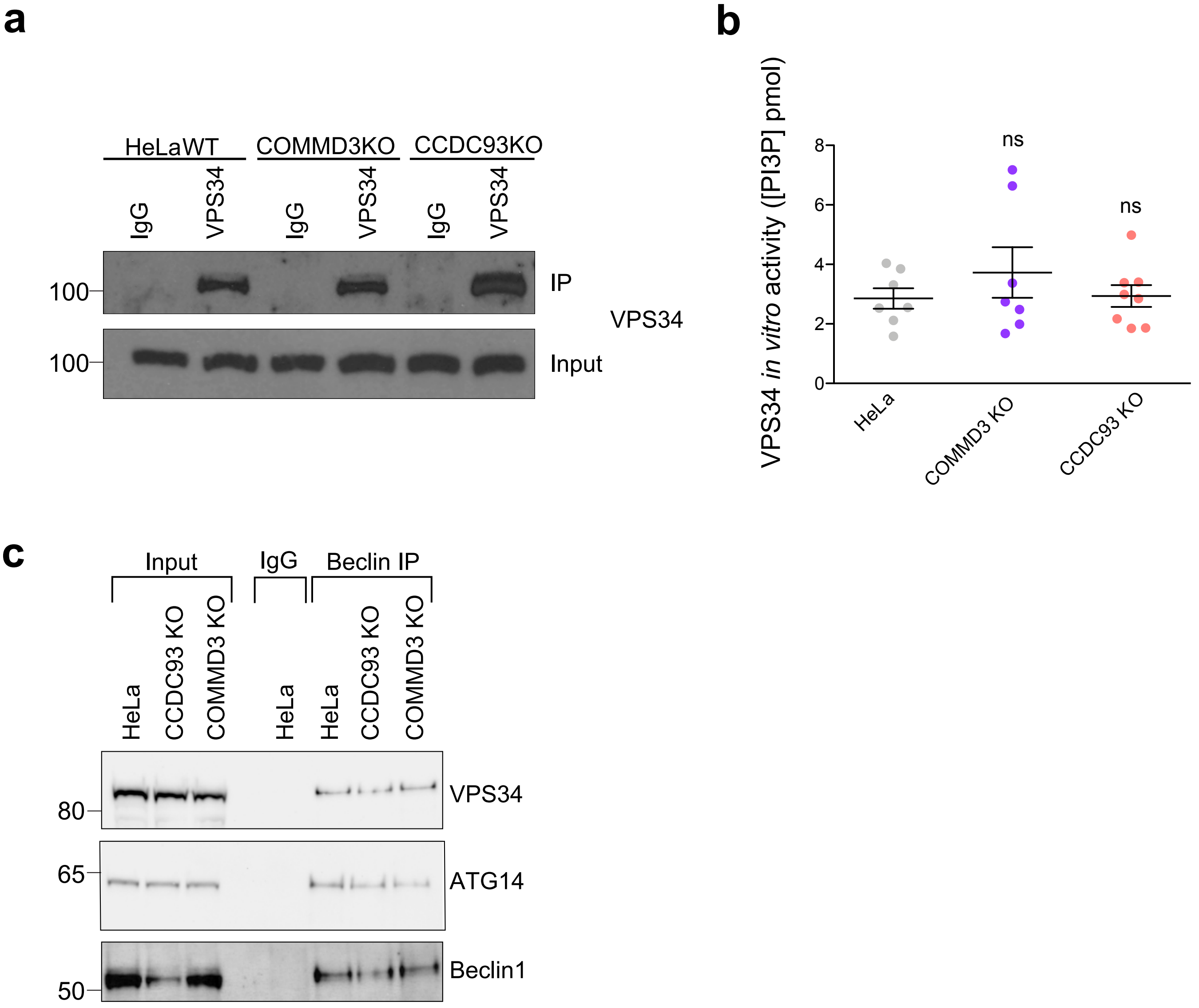


**Supplementary figure 7. VPS34 kinase activity is not increased in CCC-deficient cells. (a)** Endogenous VPS34 was immunoprecipitated from the indicated cell lines and used for *in vitro* ELISA-based VPS34 kinase activity assay. **(b)** VPS34 *in vitro* kinase activity measured in CCC-deficient cells compared to HeLa WT cells. Average of n=3 independent experiments is shown, each experiment was performed in triplicate. Values are quantified minus IgG control reaction values. The results are plotted as the mean and s.e.m for each group (one-way ANOVA and Dunnett's test to control). **(c)** Endogenous Beclin-1 was immunoprecipitated in CCC-deficient cells and the recovered material was probed for the components of VPS34 complex. Results are representative of 2 separate experiments.

# Supplementary figure 8

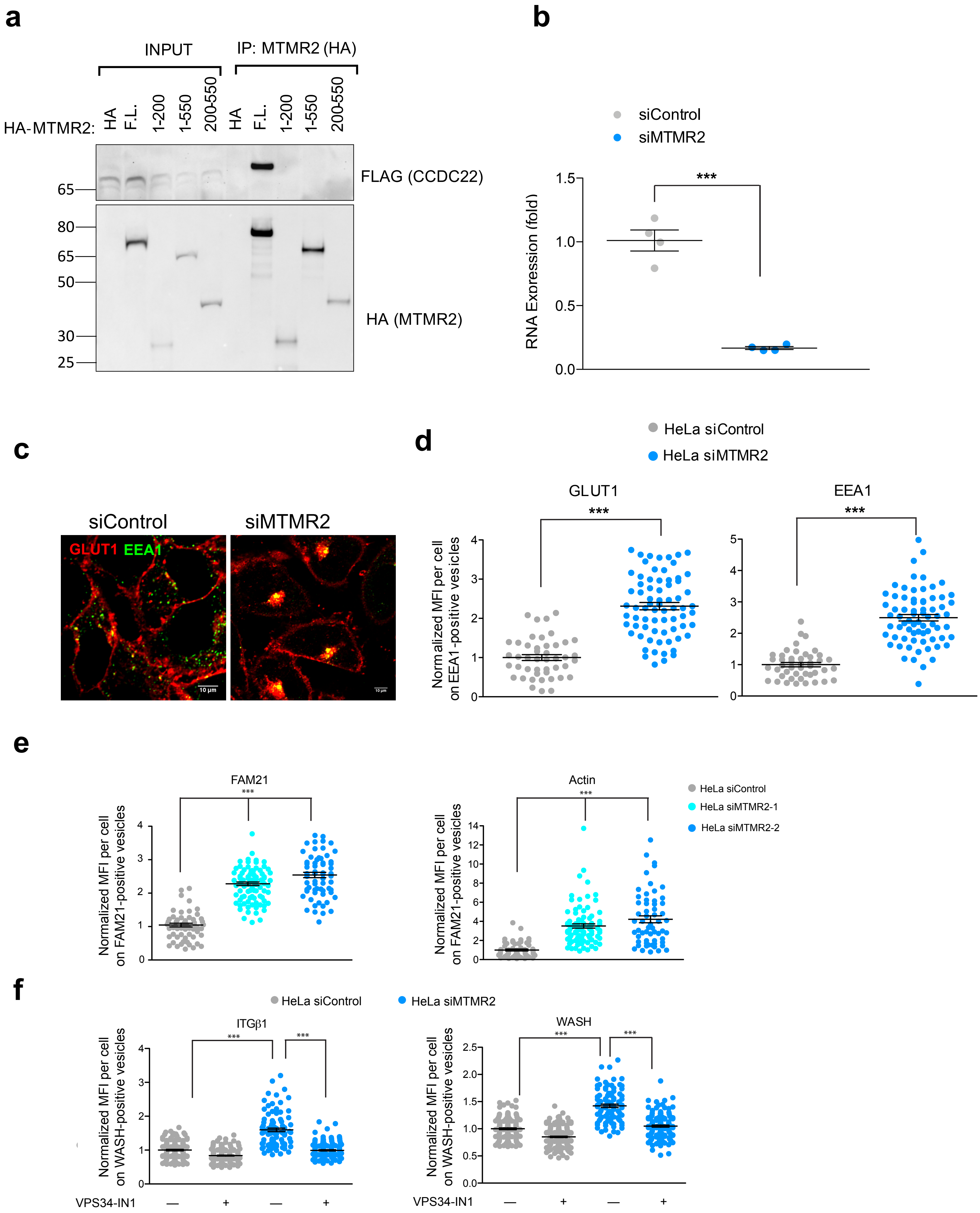

**Supplementary figure 8. MTMR2 role in endosomal recycling. (a)** The indicated constructs of 2xHA tagged MTMR2 and FLAG-CCDC22 were transfected in HEK293T cells followed by immunoprecipitation of MTMR2 using HA antibody. The recovered material was immunoblotted for CCDC22 (FLAG) and MTMR2 (HA). **(b)** mRNA expression of *MTMR2* gene in siControl and siMTMR2-transfected HeLa cells used in (c) and (d) was assessed by qRT-PCR. The results are plotted as the mean and s.e.m for each group; n=4 biological replicates; ***P<0.0001 (unpaired two-tailed t test). **(c)** Immunofluorescence staining was performed to examine GLUT1 and EEA1 localization in MTMR2 knockdown cells. Representative images are shown from 2 separate experiments, scale bar, 10 μm. **(d)** On the cells shown in (c), normalized mean fluorescence intensity of GLUT1 and EEA1 on EEA1-positive vesicles was quantified in siControl (n=45) and siMTMR2-transfected HeLa cells (n=71) and plotted as the mean and s.e.m for each group.; ****P*<0.0001 (unpaired two-tailed t test). **(e)** Quantification of normalized MFI of FAM21 per cell (left panel) and F-actin MFI on FAM21-positive vesicles (right panel) for the experiment shown in Figure 7d. Results for individual cells are plotted, along with the mean and s.e.m for each group (n=58 cells for siControl, 87 for siMTMR2-1, 63 for siMTMR2-2); ****P*<0.0001 (one-way ANOVA and Dunnett's test to control). **(f)** Quantification of normalized WASH MFI per cell (left panel) and ITGβ1 MFI on WASH-positive endosomes (right panel), corresponding to the experiment shown in Figure 7e. Results for individual cells are plotted, along with the mean and s.e.m for each group (n=150 cells for siControl, 178 for siControl VPS34-IN1, 91 for siMTMR2, 132 for siMTMR2 VPS34-IN1); ****P*<0.0001 (unpaired two-tailed t test).

# Supplementary figure 9

## Figure 1e

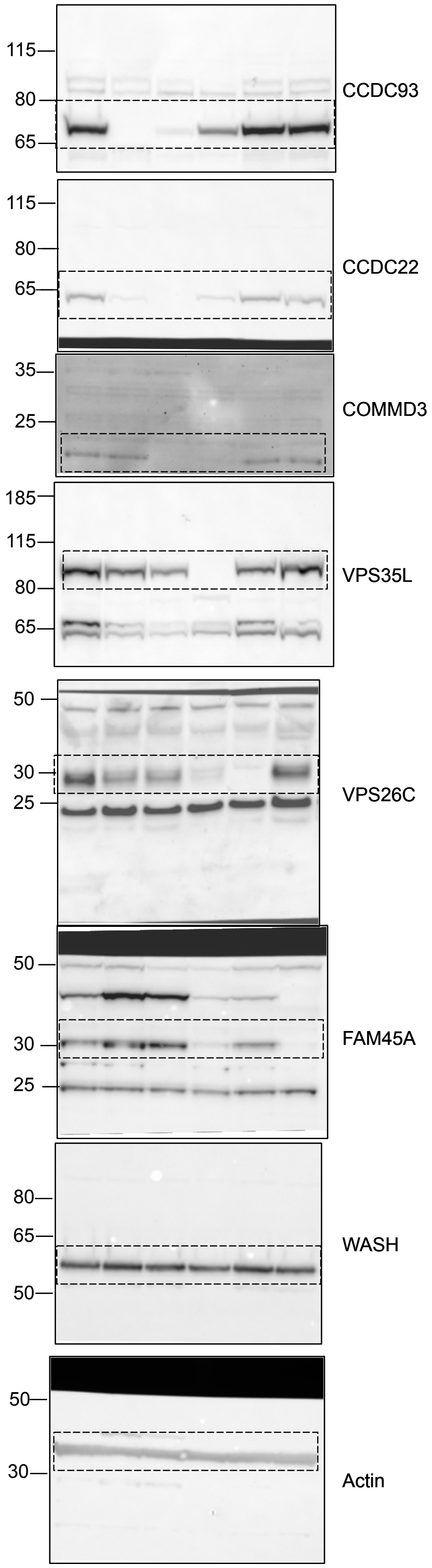


## Figure 2b

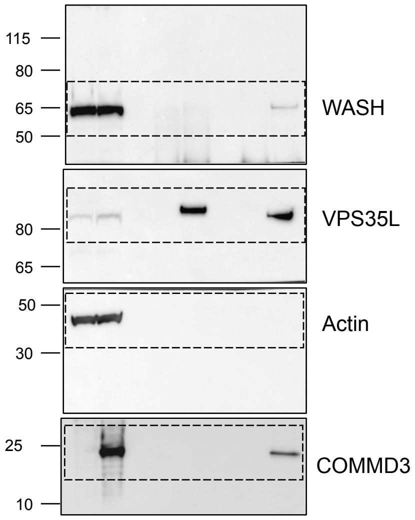


## Figure 4e

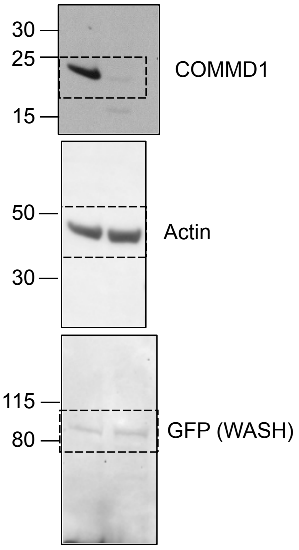


## Figure 7a

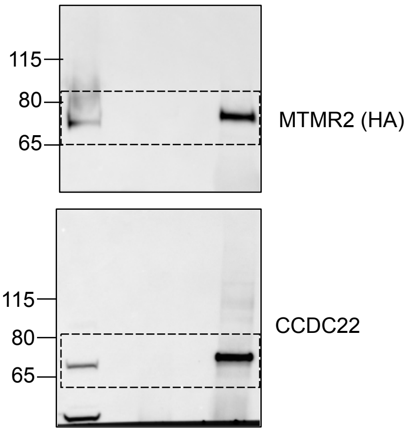


## Figure 7b

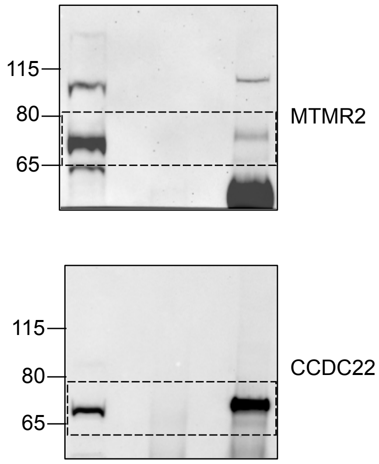

Figure 7c

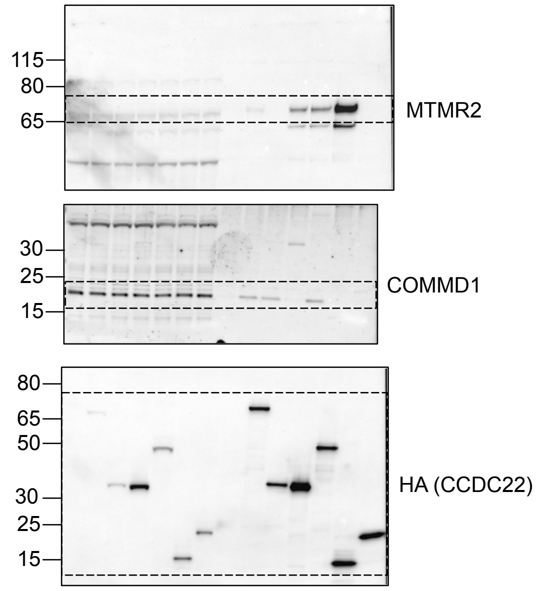


Figure 7f

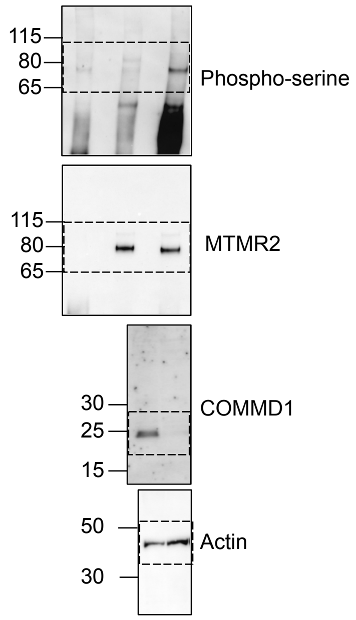


Supplementary Figure 1a

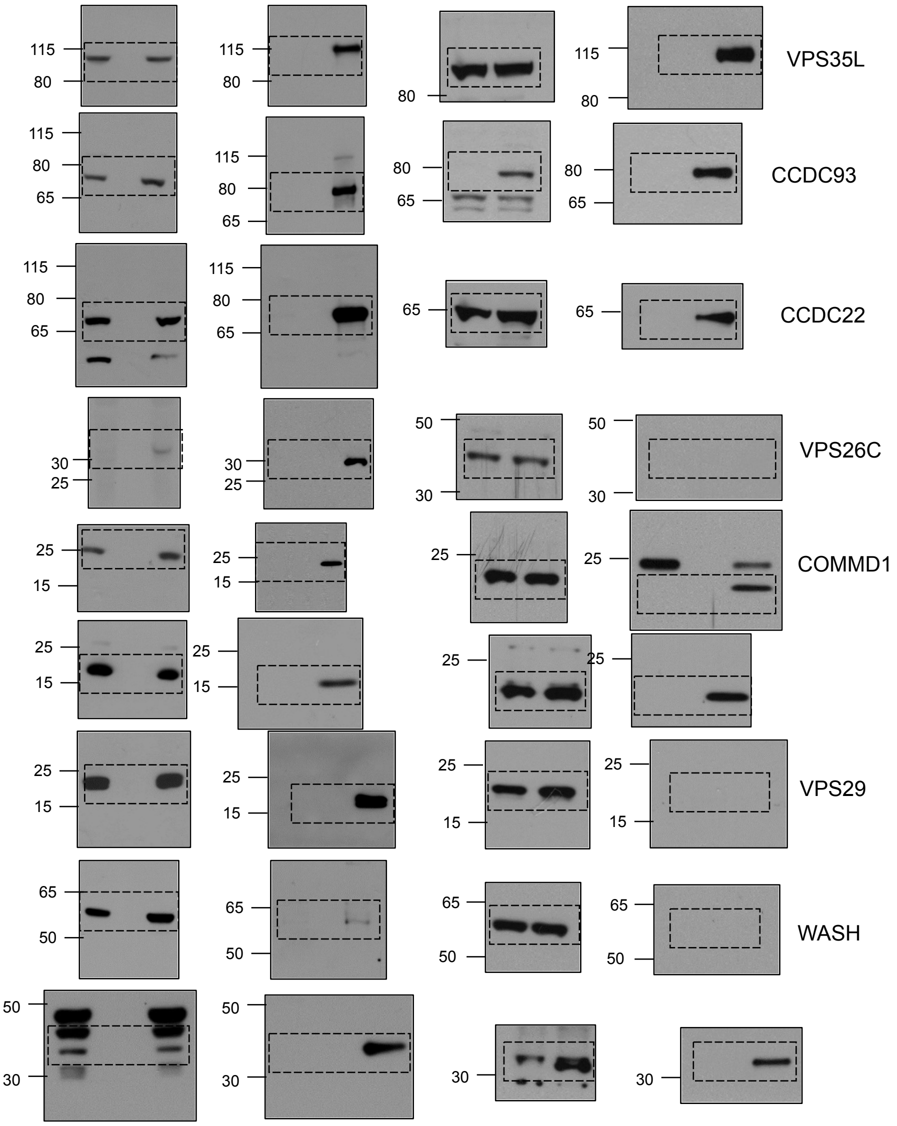


Supplementary Figure 1b

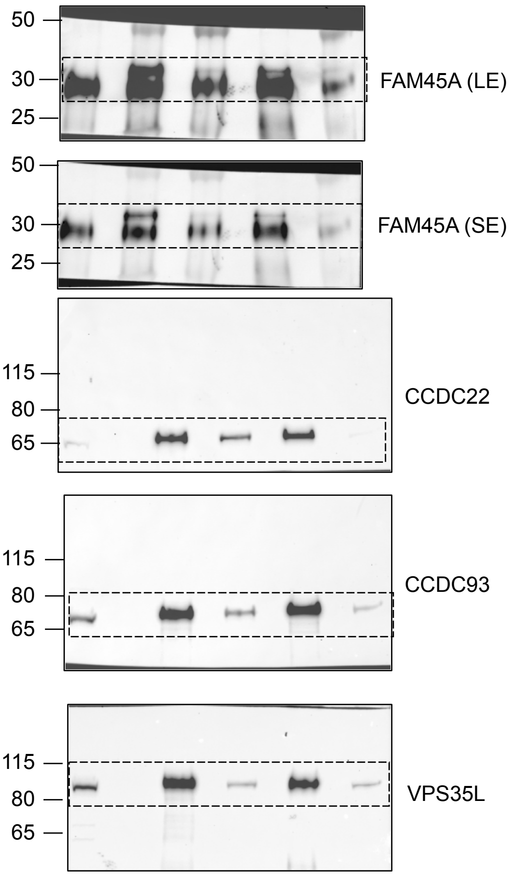

## Supplementary Figure 1d

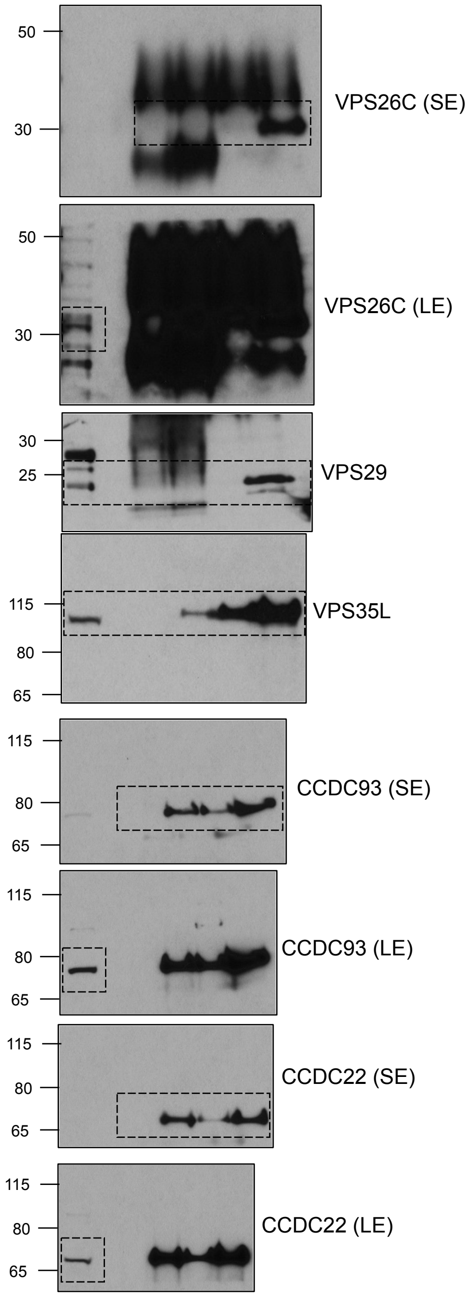


## Supplementary Figure 2c

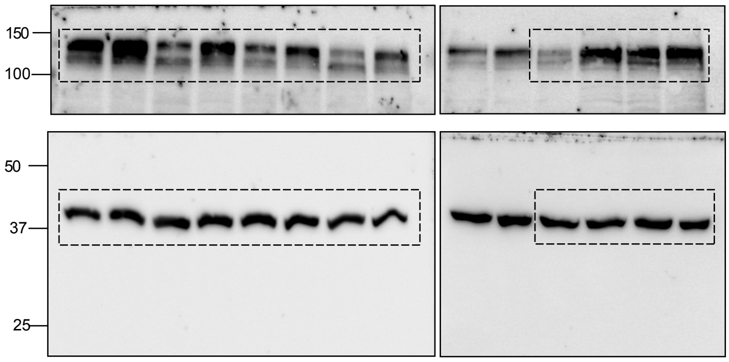


## Supplementary Figure 4d

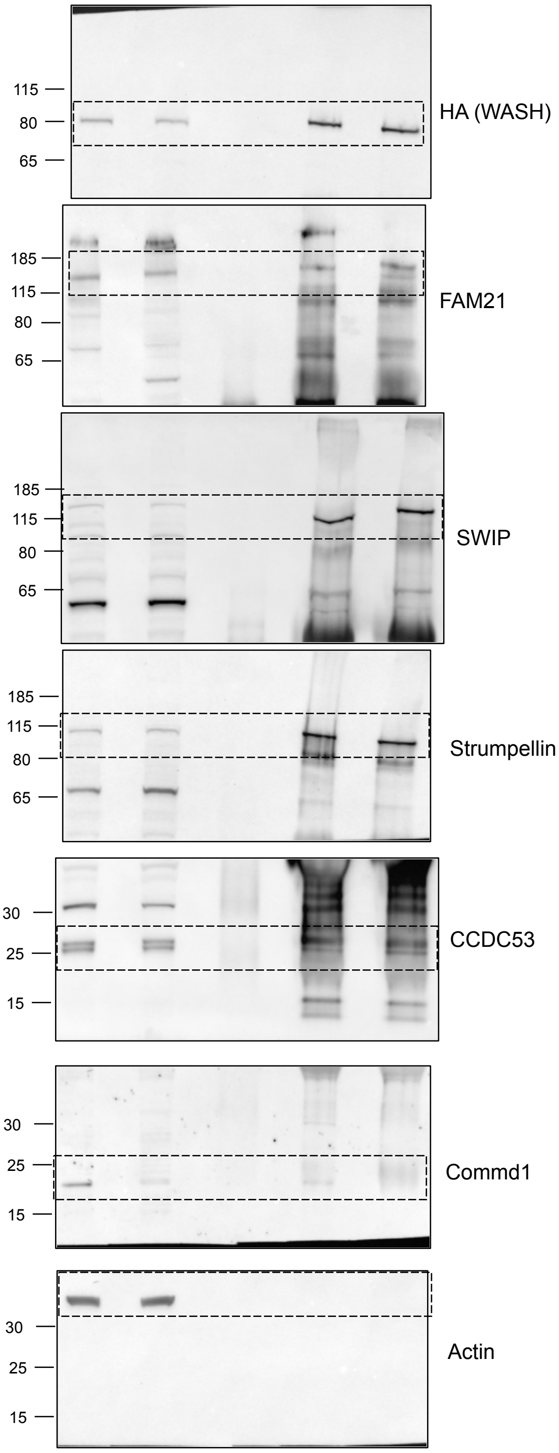

## Supplementary Figure 6d

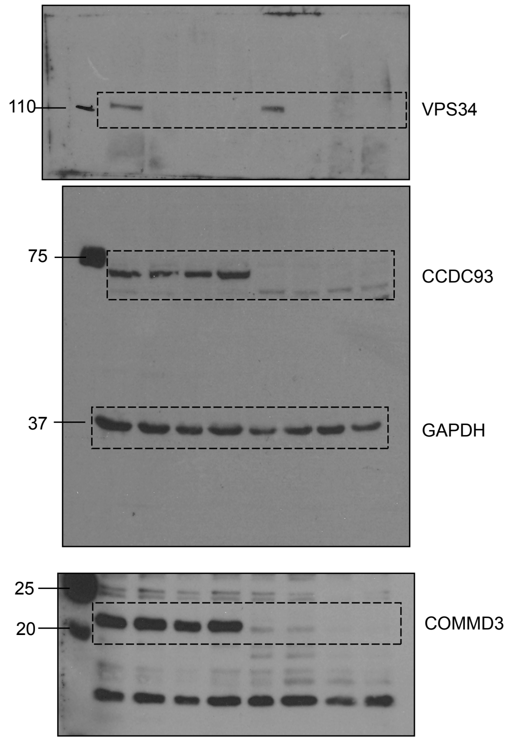


## Supplementary Figure 7c

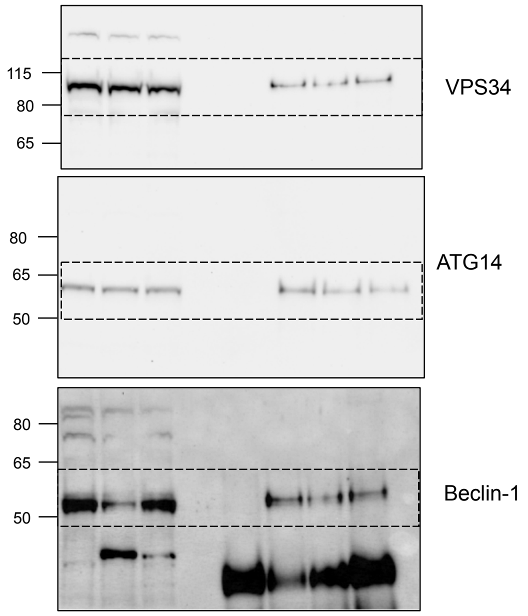


## Supplementary Figure 7a

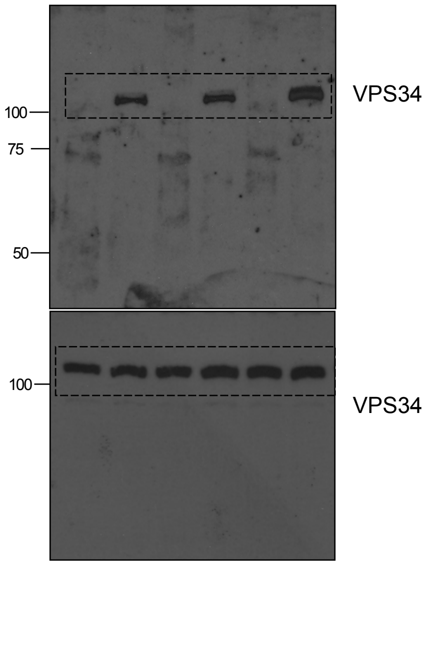


## Supplementary Figure 8a

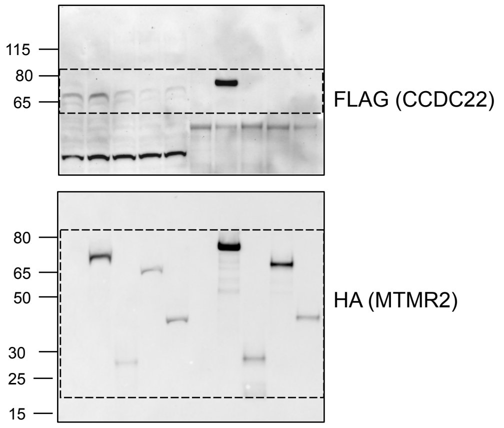


**Supplementary figure 9. Uncropped blots.** All western blot images used to derive data in this paper are shown here. SE, short exposure, LE, long exposure.

**Supplementary table 1: Targeting sequences used in shRNA and CRISPR/Cas9 experiments**

| **shRNA strategy** | | | |
|---|---|---|---|
| ***Gene target (Human)*** | ***Reference sequence*** | ***Target sequence*** | ***Reference*** |
| *COMMD1* | NM_152516 | GTCTATTGCGTCTGCAGACTT | van de Sluis et al., 2010 |
| *WASH* | NM_182905 | CCAGAGAACTACTTCTATGTG | Deng et al., 2015 |
| ***Gene target (Mouse)*** | ***Reference sequence*** | ***Target sequence*** | ***Reference*** |
| *Commd1* | NM_144514 | GATGAAGTTAAAGTCAAGCAA | This paper |
| **CRISPR/Cas9 gRNA** | | | |
| ***Gene target (Human)*** | ***Guide construct*** | ***Target sequence*** | ***Reference*** |
| *CCDC93* | Guide 1 | GACTTTCCGGCGATGTAGCTGGG | Phillips-Krawczak et al., 2015 |
| *COMMD3* | Guide 1 | CTTCACGCTTCTCCTCCGGGGTTTTA | This paper |
| *VPS35L* | Guide 1 | CACTAAGCTGAAGAACCCAC | This paper |
| *VPS26C* | Guide 1 | CACCGAATAAAGTTTATCACGCCG | McNally et al., 2017 |
| *FAM45A* | Guide 1 | CACCGTGGAGTCGGGCTGATCGGTG | This paper |

**Supplementary table 2: Antibodies used in this study**

| **Primary antibodies**<br>WB, western blot; IP, immunoprecipitation; IF, immunofluorescence staining; FC, flow cytometry; aa, amino acid. | | | |
|---|---|---|---|
| **Target** | **Source** (host species) | **Catalog or identifying number** | **Application** (dilution) |
| ARPC2 | Upstate Biotechnology | 07227 | IF (1:250) |
| ATG14 | MBL | M184-3 | WB (1:1000) |
| β-Actin | Sigma Aldrich | A5441 | WB (1:5000) |
| Beclin1 | Cell signaling | 3738S | WB (1:1000) |
| CCDC22 | ProteinTech Group | 16636-1-AP | WB (1:1000) |
| CCDC22 | Custom made (rabbit) | 711 (Ref 30) | IP (5µl per mg of protein) |
| CCDC53 | Custom made (rabbit) | MC2211 | WB (1:3000) |
| CCDC93 | ProteinTech Group | 20861-1-AP | WB (1:1000), IP (1µg per mg of protein) |
| CD29 Biotin (Integrin β-1) | Sigma | SAB4700396 | FC (1:1000) |
| COMMD1 | Custom made (rabbit) | 709 (Ref 28) | WB (1:1000), IP (5µl per mg of protein) |
| COMMD1 | Novus Biologicals | NBP2-03755 | WB (1:500) |
| COMMD3 | Abcam | ab176583 | WB (1:1000) |
| COMMD3 | Custom made (rat) | UT-R 43 (Ref 34) | WB (1:500) |
| COMMD9 | Custom made (rabbit) | 192-AP (Ref 30) | WB (1:1000) |
| Cortactin | Millipore | 05-180 | IF (1:250) |
| EEA1 | BD Biosciences | 610456 | IF (1:250) |
| FAM21 | Custom made (rabbit) | MC2188 | WB (1:3000), IF (1:1000) |
| FAM45A | Custom made (rabbit) | 95-110 (This study) | WB (1:1000) |
| FLAG | ThermoFisher Scientific | MA1-142 | WB (1:1000) |
| FLAG | Sigma | F1804 | WB (1:1000) |
| GAPDH | GeneTex | GT239 | WB (1:5000) |
| GFP | Roche | 11814460001 | WB (1:1000) |
| GLUT1 | Abcam | ab115730 | IF (1:250) |
| HA | Sigma | H3663 | IF (1:250) |
| HA | Biolegend | 901502 | WB (1:1000) |
| Integrin β-1 | Santa Cruz | Sc-13590 | IF (1:100) |
| Integrin α-5 | BD Biosciences | 555615 | IF (1:100) |
| Integrin β-1 | Cell signaling | 4706S | WB (1:1000) |
| MTMR2 | Santa Cruz | Sc-365185 | IP (1µg per mg of protein) |
| MTMR2 | Sigma | WH0008898M3 | WB (1:5000) |
| Phospho-(Ser/Thr) | Cell Signaling | 9631 | WB (1:1000) |
| Strumpellin | Custom made (rabbit) | MC2186 | WB (1:3000) |
| SWIP | Custom made (rabbit) | MC2193 | WB (1:3000) |

| | | | |
|---|---|---|---|
| VPS26A | Abcam | Ab23892 | WB (1:1000) |
| VPS26C | Millipore | ABN87 | WB (1:5000) |
| VPS29 | GeneTex | GTX104768 | WB (1:1000) |
| VPS34 | Santa Cruz | sc-365404 | WB (1:1000) |
| VPS34 | Echelon | Z-RO15 | IP (1:125) |
| VPS35 | Abcam | Ab10099 | IF (1:1000) |
| VPS35L | ThermoFisher Scientific | PA5-28553 | WB (1:1000), IF (1:250) |
| VPS35L | Custom made (rabbit) | MC2425 (This study) | IP (5µl per mg of protein) |
| WASH | Custom made (rabbit) | MC2058 | WB (1:3000), IF (1:1000) |
| **Secondary antibodies used for immunofluorescence** | | | |
| **Fluorophore** | **Source** (target species) | **Catalog number** | |
| Alexa 488 | Invitrogen (mouse) | A21202 | |
| Alexa 488 | Invitrogen (mouse) | A11029 | |
| Alexa 488 | Invitrogen (rabbit) | A21206 | |
| Alexa 488 | Invitrogen (goat) | A11055 | |
| Alexa 555 | Invitrogen (rabbit) | A21428 | |
| Alexa 568 | Invitrogen (mouse) | A10037 | |
| Alexa 568 | Invitrogen (rabbit) | A10042 | |
