# Supplementary material for: Endosomal PI(3)P regulation by the COMMD/CCDC22/CCDC93 (CCC) complex controls membrane protein recycling
Source: Nat Commun. 2019 Sep 19;10:4271. doi: 10.1038/s41467-019-12221-6 (PMC6753146; doi:10.1038/s41467-019-12221-6)
Supplement: Supplementary file 3 — Description of Additional Supplementary Files [file 41467_2019_12221_MOESM3_ESM.docx]

**Description of Supplementary Files**

**File Name: Supplementary Data 1**

**Description:** *Mass Spectrometry Results for CCDC93 and VPS26C*: Interacting partners identified by mass spectrometry after purification of CCDC93 or VPS26C.

**File Name: Supplementary Data 2**

**Description:** *Protein Interactome Database Analysis for CCC and Retriever Subunits*: Aggregate of interacting partners of CCC and Retriever subunits identified by Bioplex 2.0 and Drosophila protein interaction studies.
